# Evaluating the impacts of patient engagement on a national health research network: results of a case study of the Chronic Pain Network

**DOI:** 10.1186/s40900-023-00491-w

**Published:** 2023-08-30

**Authors:** Laura Tripp, Dawn P. Richards, Jennifer Daly-Cyr, Therese Lane, Delane Linkiewich, Kimberly N. Begley, Norman Buckley, Maria Hudspith, Patricia Poulin, Julia Abelson

**Affiliations:** 1 Public and Patient Engagement Collaborative, McMaster University, Hamilton, ON Canada; 2 Chronic Pain Network, McMaster University, Hamilton, ON Canada; 3 Five02 Labs Inc, Toronto, ON Canada; 4 Department of Psychology, University of Guelph, Guelph, ON Canada; 5 Department of Anesthesia, Faculty of Health Sciences, McMaster University, Hamilton, ON Canada; 6 Pain BC, Vancouver, BC Canada; 7 The Ottawa Hospital Pain Clinic, Ottawa, ON Canada; 8 The Ottawa Hospital Research Institute, Ottawa, ON Canada; 9 Department of Anesthesiology and Pain Medicine, Faculty of Medicine, University of Ottawa, Ottawa, ON Canada; 10 Department of Health Research Methods, Evidence and Impact, McMaster University, Hamilton, ON Canada

**Keywords:** Evaluation, Patient and public involvement, Patient engagement

## Abstract

**Background:**

The Chronic Pain Network (CPN) is a pan-Canadian research network focused on innovating and improving the quality and delivery of pain prevention, assessment, management and research for all Canadians. An important focus of the CPN is to work in collaboration with patient partners. Patient partners, researchers and clinicians work together in all aspects of the research network including on funded research projects and in the governance of the Network. Given this focus, the CPN identified the importance of evaluating their patient engagement work to understand its functioning and impact.

**Methods:**

The objective of this exploratory evaluation case study was to understand the impacts of patient engagement on the CPN. The CPN worked with an external evaluation team which established an arms-length approach to the evaluation. Interviews were conducted with CPN members, including patient partners, leadership, funded researchers and committee co-chairs, at three discrete time points to trace the evolution of the patient engagement program within the Network. Key Network documents were also collected and reviewed. Data were analyzed following each set of interviews using content analysis guided by the principles of constant comparison and qualitative description. A final round of analysis was conducted using the Engage with Impact Toolkit, an impact measurement framework, to identify impacts of engagement.

**Results:**

Impacts of patient engagement were identified at the individual, network, funded research project and research community levels. These impacts were observed in the following areas: (1) building community; (2) developing knowledge, skills and resources; (3) increasing confidence; (4) influencing priorities and decisions; (5) enabling additional opportunities; (6) promoting culture change; and, (7) coping with experiences of living with chronic pain.

**Conclusions:**

While not without challenges, the patient engagement efforts of the CPN demonstrates the impact engaging patient partners can have on a national research network and related policy activities. Understanding the approaches to, and impacts of, patient engagement on health research networks can illuminate the value of having patient partners engaged in all aspects of a research network and should serve as encouragement to others who look to take on similar work.

**Supplementary Information:**

The online version contains supplementary material available at 10.1186/s40900-023-00491-w.

## Abstract (plain language summary)

The Chronic Pain Network (CPN) is one of a group of research networks that was funded by the Canadian Institutes of Health Research (CIHR) to support patient-oriented research in chronic diseases. From the beginning of its work, the CPN has included patients as partners. Patient partners are co-chairs of all Network governance committees, funded projects are required to include patient partners in their work and there is a committee dedicated to engagement, the Patient Engagement (PE) committee. The PE Committee determined that it was important to evaluate how the CPN was engaging with patient partners and collaborated with the Public and Patient Engagement Collaborative (PPEC) to evaluate this work. The PPEC, along with members of the PE Committee, identified understanding the impact of patient engagement as an important part of the evaluation. This paper provides a description of the impacts of patient engagement on the people who were involved in the CPN, on the CPN’s work and way of being, and on the broader pain research community. Based on the results from three sets of interviews and review of Network documents, we share impacts identified in seven areas: (1) building community; (2) developing knowledge, skills and resources; (3) increasing confidence; (4) influencing priorities and decisions; (5) enabling additional opportunities; (6) promoting culture change; and, (7) coping with experiences of living with chronic pain. This research shows us the impact that engaging patient partners can have on a national research network, and the areas where greater focus could, perhaps, lead to even greater impacts in future networks.

**Supplementary Information:**

The online version contains supplementary material available at 10.1186/s40900-023-00491-w.

## Background

The concept of “nothing about us without us” has taken hold in the research field as increasingly patients, families and caregivers are collaborating with researchers and research organizations to guide, support and influence research as partners rather than as research participants. This movement toward patient partnership has been seen in Canada through the development of the Canadian Institutes of Health Research’s (CIHR) Strategy for Patient-Oriented Research (SPOR) [1] and internationally through organizations such as the Patient-Centered Outcomes Research Institute (PCORI) [2] in the United States and the National Institute for Health and Care Research (NIHR) [3] in the U.K. The patient partner literature documents a large and rich set of experiences of patient partners engaging in different types of research activities and teams [4]. As patient engagement in research has grown, so has the call for patient partners to be engaged in all phases of the research process and more strategically in the oversight and governance of research itself. Including patient partners in research networks is one way that this has been achieved; by including them at the level of setting priorities for the research being carried out and its funding, patient partners are able to influence earlier stages of research. The inclusion of patient partners in research networks has been well documented both in Canada [5–7] and internationally [8]. There is, however, an ongoing need to evaluate these partnerships and their impacts. To address this need, an exploratory evaluation case study was conducted to understand the impacts of patient engagement on a pan-Canadian research network focused on chronic pain, the Chronic Pain Network (CPN). The overall objectives of the evaluation were two-fold: (1) to evaluate the structures and processes for engaging patient partners in the CPN and to provide input into how these could be improved, and (2) to understand the impacts of the CPN’s patient engagement efforts. This paper presents the results for the second objective, the impacts of patient engagement on the CPN.

### Chronic Pain Network

The CPN was one of five chronic disease research networks competitively funded by CIHR’s SPOR initiative to support patient-oriented research in chronic diseases. Patient partners were part of the earliest conversations about its inception when CIHR announced their call for proposals in October 2014, and played a pivotal role in priority setting, proposal development, governance and conduct of research throughout the entire first phase of the CPN, which ran from January 2016–March 2022. The CPN has since received further funding from CIHR to continue and expand its work, now focusing on knowledge mobilization and implementation. Phase 2 of the CPN will run from April 2022–March 2026.

Phase 1 of the Network aimed to innovate and improve the quality and delivery of pain prevention, assessment, management and research for all Canadians. Importantly, a central focus of the CPN was collaboration with patient partners. Patients, researchers and clinicians worked together in all aspects of the research process (e.g., patients partnering on research studies funded by the Network) and Network functioning (e.g., patient partners were members and co-chairs of the CPN’s governance committees). The CPN uses the CIHR definition of patient engagement to inform its work, highlighting that engagement is about “*meaningful collaboration…[that] helps to ensure that research being conducted is relevant and valuable to the patients that it affects”* [9].

The first phase of the CPN included 25 national partners with 22 Network-funded research projects led by a coordinating centre team at McMaster University in Hamilton, Ontario, Canada. Over the course of the first phase of the Network, 22 patient partners were involved at the Network level. The Network governance included six committees, each of which had at least one patient partner co-chair in addition to other patient partner members. All CPN-funded research projects were required to engage with a patient partner on their study team and to report on these engagement activities.

The patient engagement strategy was overseen and guided by the Patient Engagement (PE) committee, a committee of patient partners, researchers, CPN leaders, and staff who provided guidance, support and leadership to patient engagement within the Network. The original mandate of the PE committee was to “*ensure meaningful engagement of diverse patients with chronic pain in the Governance and Committee structures and projects of the Network*” while also aiming to increase the capacity of “*network members to meaningfully engage patients in their research projects*” and of “*patients to be engaged in the network*” [10]. Over time this mandate grew to focus more broadly on influencing the broader pain research ecosystem in Canada.

The CPN PE Committee identified a desire to evaluate the implementation of the Network’s patient engagement strategy and approached the Public and Patient Engagement Collaborative (PPEC) at McMaster University to act as the external evaluators. The PPEC developed an overall approach to the evaluation in collaboration with the PE Committee, who served as a steering committee for the evaluation, helping to identify the overall focus for the evaluation as well as specific phases of data collection based on interim findings (e.g., a decision to focus on research projects in round 2 interviews). PE Committee members provided reflective comments on interim results presented to them but did not participate directly in any data collection activities (as researchers), in data analysis or in the initial interpretation of results.

## Methods

An evaluation of the CPN was conducted from 2018 – 2022. Given that little work has been done to date to understand the impacts of patient engagement on health research networks, an exploratory evaluation case study approach was taken [11]. This single case exploratory study examined the CPN as a whole, with a focus on how engagement unfolded within the CPN governance and committee structure and at the CPN-funded research project level in relation to the overall Network and its supporting infrastructure. While the overall goal of the evaluation was to assess both process and impact, the research presented in this manuscript is focused on the impact of patient engagement in the research network. The research question for the impact evaluation was: What are the positive and negative, intended and unintended impacts of the patient engagement strategy on the CPN, as well as on those engaged in the Network and the broader Canadian pain research community? [12]. The Engage with Impact Toolkit informed the development of this question, highlighting the importance of considering impacts at multiple levels (on people, programs, organizations and systems) [13].

The impact evaluation drew on two main data sources: (1) document reviews and (2) key informant interviews. Data collection occurred between September 2018 and March 2022 to cover different phases of the CPN activities. The findings from the interviews and document review were integrated throughout the data collection and analysis phases to allow documents to inform interviews (e.g., areas of focus, potential impacts/outcomes to address) and to contextualize findings during analysis.

### Document review

Key documents related to patient engagement within the CPN were reviewed to provide context to the case study and to identify instances where the impacts of patient engagement were indicated. Documents reviewed included CPN internal documents (e.g., committee agendas, grant application) and publicly available documents (e.g., newsletters, annual reports, publications). Additional documents identified by interviewees were collected and reviewed (e.g., publications, knowledge translation products). Documents were analyzed using content analysis to identify examples of the impacts of patient engagement and to provide further context on the CPN’s patient engagement activities.

### Interviews

Three rounds of semi-structured interviews were conducted that roughly traced the evolution of patient engagement within the CPN. Time points were selected to allow for evaluation results to inform the Network’s activities over time while attempting to minimize the burden on respondents. Interviews were conducted at the beginning of the evaluation (Fall 2018), at the mid-point (Winter 2020) and at the end of the Network’s initial funding period (Winter 2022). Each round of interviews focused on a different aspect of the CPN’s engagement work which resulted in different sampling frames for each (Table 1). Interview guides were structured around the objectives for each component of the evaluation (e.g., process and impact outcomes) and informed by areas of interest identified by the PE committee, shaped by the review of interim results (See Table 1). For example, the results of the first round of interviews suggested more information was needed on the progress of the CPN funded research projects’ engagement efforts, and thus this was a focus for the second round of interviews. The PE Committee provided feedback on the proposed focus of the interviews but were not involved in the development of the guides themselves. Interview guides were informed by those used in similar studies of patient engagement in research [14, 15] and from relevant evaluation tools and toolkits [13, 16–18]. Interview guides for all three rounds of interviews are included in additional file 1.

**Table 1:** Data collection approach

Time	Interview focus	CPN groups invited to participate	Number of interviews/Number invited*
Fall 2018	The CPN’s approach to engagement (governance structure, engagement in funded research projects); initial experiences with the CPN; challenges and successes to date	Committee co-chairs CPN Leadership Highly involved network members	14/23
Winter 2020	Challenges and successes of patient engagement within the CPN; engagement within funded research projects; initial impacts of the CPN’s engagement approach	Patient partners CPN-funded researchers	19/50
Winter 2022	Challenges and successes of patient engagement within the CPN; next steps for the CPN and patient engagement generally; the impacts of the CPN’s engagement activities	Patient partners PE Committee members CPN Leadership	13/15

*Some participants participated in multiple rounds of interviews, thus these numbers do not represent unique individuals across interview phases

Inclusion criteria for potential key informants for each round were developed based on the focus of the evaluation phase and the goals of the exploratory case study (Table 1). The concept of information power informed sample size decisions [19]. The narrow aim of the study, the specificity of the sample, the focus on a single case and the depth of the interviews suggested the sample had a high level of information power and thus a smaller sample was required. The CPN provided the PPEC research team with the names and contact information for all potential interviewees who met these inclusion criteria. All interviewees were associated with the CPN, either as staff members, committee / governance members (researchers and patient partners), or as funded researchers. Inclusion criteria focused solely on the role of individuals within the network (Table 1). Emails were sent to potential participants by the PPEC to share more information about the study and assess interest in participation. If individuals agreed to participate in the study, interviews were scheduled at a time that was convenient for them.

The study was reviewed and approved by the Hamilton Integrated Research Ethics Board (HIREB, Application # 5039). Interview participants provided written informed consent prior to their first interview. Participants were invited to make decisions regarding potential participation at each interview time point—participating (or not) in one round of interviews did not mean individuals were required (or unable) to participate in future rounds, should they be eligible. The key aspects of the consent process were reviewed prior to each subsequent interview to ensure ongoing consent to participate.

Interviews were up to 60 min in length and were conducted either using a teleconference line or virtually using Zoom. Interviews were conducted by a PPEC Research Coordinator or Research Assistant. Interviews were recorded and transcribed verbatim. All interviews were confidential and CPN staff and members were not informed of who had participated in the interviews and who had not. Transcripts were analyzed using content analysis by members of the evaluation research team (LT, JA, and research assistants) guided by the principles of constant comparison and qualitative description following each set of interviews [20–22]. A final round of analysis was conducted at the end of the evaluation to focus on the overall impacts of the patient engagement strategy, initially reviewing the analysis already completed, followed by an additional round of analysis to tease out specific impacts. A deductive content analysis approach was taken, and the Engage with Impact Toolkit was used to develop the initial analytical framework [13]. The Engage with Impact Toolkit identifies eight conceptual domains of impact for patient engagement, each of which formed one component of the analytical framework. The analysis allowed for additional domains to emerge inductively during the analysis process. NVivo 12 was used to support qualitative data analysis and for data management (QSR International, 2019). The results of the impact analysis were presented to the PE Committee members as well as the members of the authorship group, some of whom may have been eligible to participate in the interviews. While this process did not lead to any changes in the findings, it allowed for further contextualization of the results.

### Patient partner involvement

This research was conducted in collaboration with patient partners, and other stakeholders from the CPN. The evaluation as a whole was guided by the PE Committee whose mandate included to “*evaluate and report on the outcomes of patient engagement in Network activities*” [10]. The PE Committee collaborated with the PPEC research team to determine the areas of focus for the evaluation, to interpret the evaluation results and to consider next steps.

A subgroup of the PE Committee came together for discussions regarding how the results of the evaluation would be shared publicly. Patient partners, CPN staff and researchers contributed to these discussions, to the decision to write this manuscript and to its development. The Guidance for Reporting Involvement of Patients and Public (GRIPP-2) Long Form was used to guide the reporting of the involvement of patient partners in our research and is included as additional file 2.

## Results

A total of 46 interviews were completed across the three time periods, with 28 individuals. Individual respondents participated in up to three interviews each, with the majority participating in one (42.9%, n = 12) or two (46.4%, n = 13) interviews. Just over half (53.6%, n = 15) of the interviewees were patient partners. The remaining participants were researchers (32.1%, n = 9) or CPN leadership/staff (14.3%, n = 4). The interviewees offered rich insights into the impacts of patient engagement on the activities of the CPN and the broader pain research community in Canada. The Engage with Impact Toolkit was used to structure the results and the impact domains [13]. Four of the domains from the toolkit were seen in the impacts achieved by the CPN. These included: (1) developing knowledge, skills and resources; (2) increasing confidence; (3) influencing priorities and decisions; and, (4) promoting culture change. The remaining domains from the toolkit (patient outcome and experience; effectiveness and efficiency; patient centredness; equity and inclusivity) were not identified as impacts of the CPN in the data. Equity and inclusivity did appear in the data as an area of future growth. An additional three themes were identified that were not included in the Toolkit. These include: (1) building community; (2) enabling additional opportunities; and, (3) coping with experiences of living with chronic pain. Each of these themes is explored below, including challenges faced in each.

### Building community

The CPN was, above all else, a Network of patient partners, researchers and others who came together with a common goal of improving the lives of people living with chronic pain. As a result, one of the most significant impacts of the patient engagement strategy of the Network was the development of a community of individuals with a shared interest in chronic pain: *“that’s what patient engagement is all about, is for the patients to ensure that other people understand the patient’s point of view and for [patients] to understand how the researchers work…we can bring the two of them together and we can become a pretty serious team”* (Interview #2022-03: Patient Partner)*.*

Several strategies were successfully used to build this sense of community and collaboration over the first phase of the Network. One key strategy was the CPN’s governance structure which ensured that all CPN committees were co-chaired by a researcher/staff member and a patient partner. While this structure had several benefits and challenges, one noted benefit was increased awareness of all members of the Network and their roles: *“having patients involved at the governance level really does help…they’re aware of the researchers and the researchers are aware of them”* (Interview #2022-01: Patient Partner).

Beyond specific structural approaches, community was developed using several engagement strategies. Given the national scope of the Network, virtual meetings were used from the outset. Prior to the COVID-19 pandemic, however, periodic in-person meetings (usually annually) were held which helped bring together a variety of stakeholders to make connections: *“to actually sit and have a coffee or chat with people at tables that were patient partners…I think that was probably the best thing for me”* (Interview #2018-13: Researcher).

CPN members highlighted how their experiences with the Network helped them to build a professional and personal community. These connections led to both interpersonal connections and friendships: *“I think that one of the greatest benefits…was to meet all these people…and appreciate them, both as individuals personally and professionally as well. So that was very enriching for me and something I will cherish forever”* (Interview #2022-03: Patient Partner). The CPN was formed at a time when there was no national pain research organization, and thus the Network’s engagement efforts also contributed to the development of a community where one was lacking: *“[CPN] brought together a community of chronic pain patients from across the country where it never existed before”* (Interview #2020-04: Patient Partner).

The sense of community was especially strong amongst the patient partners themselves, an unanticipated but highly valued output: *“There’s less attention [in engagement literature] paid to the creation of community among people who live with a particular condition…. what we’ve seen over the Network is this really incredible community building happen[ing] among the people with lived experience…we didn’t articulate [that] as a goal….I’ve seen this as an incredible outcome”* (Interview #2022-11: CPN Staff/Leadership). The COVID-19 pandemic was a time of stress for many, especially those in the chronic pain community. During this time, periodic check-ins were set up for patient partners to connect with each other about non-CPN topics. While initially these were facilitated by a CPN staff member, after the first few meetings this facilitating shifted to the patient partners who took turns hosting. This sense of community helped some patient partners to feel less isolated: *“I made connections all over Canada…something I would have never done before. I think with chronic illness, you’re very isolated, and this was just so wonderful for me”* (Interview #2022-12: Patient Partner).

Despite the general sense of community amongst CPN members, these impacts were not felt universally. A small number of patient partners, mainly those who were not members of the PE Committee, conveyed a sense of not being included with the *“core group of patient partners*” and that their ability to contribute to the Network and feel the impact of their contribution was limited by this. Also, many Network members highlighted the lack of diversity within the Network as a challenge that may have limited the impact of the Network: *“we don’t have a diversity of patient partners in terms of visible minorities, indigenous people….I think the people we have are fairly high or mid-SES…we don’t have a lot of people from low-income areas….I wish we could have done a little bit better in terms of reaching out to other minority populations and hear their voice”* (Interview #2018-13: Researcher). Further, while the Network’s patient partners did have a variety of experiences with chronic pain, there could have been greater diversity in the types of chronic pain experiences and ages represented. This was acknowledged early in the evaluation, but the Network was unsure how to address it, given the challenges of bringing new members into an existing structure and the uncertainty of the ongoing sustainability of the Network at the time. This is a focus for the next phase of the Network, currently underway [23].

### Developing knowledge, skills and resources

The CPN’s approach to patient engagement contributed to knowledge and skill development around patient engagement and research more generally. This occurred in several ways throughout the Network including specific educational opportunities related to engagement and through opportunities to apply these skills through Network activities.

Participating in the Network provided an opportunity for researchers and patient partners to learn and develop new patient engagement skills. While there were a number of challenges when it came to engaging patient partners in CPN funded research, several researchers found their footing in patient engagement through the Network and changed their approach as a result: *“[researchers started off] floundering, figuring out “how am I going to engage with patients?”…to now reaching out to patient groups on their own, going out to the community and engaging with community groups. That just complete total shift in their mindset about the value of engaging patients”* (Interview #2022-02: Patient Partner). Patient partners also spoke of being unclear at the outset as to what was involved with engagement and learning over the Network’s mandate: *“Patient engagement was very new to me…the more time I spent learning from all my peers and colleagues, the more time I spent with the Network, the more I felt prepared”* (Interview #2020-01: Patient Partner). In addition, patient partners’ participation led to increased knowledge about research, the research funding process and advocacy. Mentorship played an important role in this process: *“I met so many amazing advocates who have taught me so much about using your lived experiences to make change and to change how things are being done”* (Interview #2022-10: Patient Partner).

Resources and supports developed by the CPN also had an impact on those outside of the Network: *“the amount of resources we’ve created, or not necessarily physical resources…but also things like webinars…to teach about patient engagement…have really impacted how people in the pain space see patient engagement”* (Interview #2022-10: Patient Partner). More formal resources were created contributing to the science of patient engagement broadly. Members of the Network, including patient partners, came together to form a working group and wrote a manuscript on authorship and acknowledgement in patient-oriented research [24, 25]. This manuscript has been widely used with 10,000 accesses, 25 citations and 512 mentions on twitter as of August 2023 [26]. CPN staff and patient partners also worked with other SPOR Networks in chronic disease and the national Primary and Integrated Health Care Innovations (PIHCI) Network to develop recommendations on patient engagement compensation [27]. Towards the end of the first phase of the Network, a group of patient partners came together to reflect on patient engagement and its outcomes through the first phase of the network and put their learnings into a publicly available video [28]. Further, a researcher approached the PE Committee to collaborate on a survey of trainees conducting pain research in Canada’s perspectives and experiences with patient engagement [29]. This survey further advanced knowledge related to engagement in the Canadian pain research community more broadly. These activities pushed the science of engagement, which as one participant noted, was an unexpected impact: *“I find it really interesting that the group has contributed to the science of patient engagement, not just the science of pain”* (Interview #2022-13: CPN Staff/Leadership).

### Increasing confidence

Numerous patient partners and researchers attributed their increased levels of confidence to their participation in patient engagement activities within the Network. Members reflected on how the experience of being a CPN member had an impact on their overall confidence level: *“I mean it brought me out of my shell, it really did….”* (Interview #2022-12: Patient Partner). The increased confidence was not only identified by individuals, but by others in the Network: “*At the beginning, some patient partners were…not really speaking up, but as they got involved in the Network and realized…their role, I think now everyone feels quite comfortable speaking up”* (Interview #2020-10: Researcher). For some, this increased confidence led to members taking on more roles within the research community: “*a number of [patient partners] came from not really knowing anything about research, not being very confident in terms of speaking up, to being people who are now on multiple research teams. They’ve given multiple presentations at national conferences and webinars”* (Interview #2022-13: CPN Staff/Leadership).

Those researchers and patient partners who were more experienced with patient engagement mentored others: “*A lot of people came in [to the CPN] knowing about patient engagement, knowing about its value and worth, but have really shared that passion for patient engagement with others. And it’s really been infectious…and for the people who already knew about patient engagement, really strengthening that passion”* (Interview #2022-10: Patient Partner). These experienced patient partners spoke of the gratification they found in seeing the less experienced patient partners learning and increasing their confidence over the course of the Network: “*[new patient partners] have learned and grown and found their voices…[and] are becoming leaders in their own right…it’s wonderful to see the confidence levels of those individuals increase with experience”* (Interview #2022-09: Patient Partner). Researchers and other CPN members also highlighted the impact that their role in the Network had in increasing their confidence in their ability to engage patient partners in their work.

### Influencing priorities and decisions

Patient partners influenced the priorities and decisions of the Network from the very outset. In parallel, as the Network was being established, network members, including patient partners, engaged in a national adult pain research priority setting exercise, with patient partners included at all levels of the research including on the steering committee and patient advisory committee [30]. This informed decisions about what research the CPN would fund and undertake. Subsequently a separate exercise was also undertaken to determine the priorities relation to pediatric chronic pain. Those with lived experience with pediatric chronic pain and caregivers to those with pediatric chronic pain were full collaborators in the exercise [31]. Again, these findings informed the Network’s activities.

The impacts that patient engagement had on Network priorities grew over time as the Network evolved and the patient engagement strategy matured. The Network took on several new activities and projects related to patient engagement that had not been included in initial planning, such as the paper on authorship with patient partners [24], that occurred as a direct result of the Network’s patient engagement approach. A patient partner described how this came about: *“It was an idea I had brought up regarding authorship….that quickly became a little sub-committee working on this authorship paper….it was a lot of work for folks like me…I knew it wouldn’t be simple, but I guess it had to be done….it provides a guideline for both researchers and patient partners to understand authorship and what it takes in a scientific journal”* (Interview #2020-17: Patient Partner). The governance structure of CPN itself, which places a patient partner in the co-chair role with a researcher or staff member, was also viewed as providing opportunities for patient partners to influence Network priorities. While there was variation across the committees with respect to the extent to which these leadership roles were able to shape the priorities and directions of the Network, this co-chair approach was broadly viewed as providing an effective vehicle for patient partners to shape the Network’s work and decisions. In early evaluation interviews, issues surrounding the co-chair model came up more frequently with concerns that patient partners were *“token co-chairs”* and their perspectives were not equally considered by all members of the committee. This appeared to shift over time as the culture of engagement became more robust within the Network, but some challenges remained. It was noted by some that the impact that patient partners had on committees’ priorities and decisions were, in some cases, different: *“[patient partner input] happens everywhere, just in some committees it happens a bit more…due to the nature of the committee and the tasks that are involved in that committee”* (Interview #2020-10: Researcher). Despite the challenges, however, the co-chair model was positively viewed. As one member noted: *“this concept of co-governance where patient partners are throughout all the committees as co-chairs, I think that’s been fairly impactful…as long as their other co-chair is particularly supportive, it can’t help but change the outcomes of what the committees are doing”* (Interview #2022-13: CPN Staff/Leadership).

Patient engagement was a requirement for all CPN funded studies and was assessed through annual reporting to the Patient Oriented Research Committee [10] which was *“responsible for reviewing each of these [funded] projects and saying, “Okay, where is your evidence of patient engagement?”* (Interview #2020-12: CPN Staff/Leadership). There were challenges with how patient partners were engaged within the funded research projects and this was a learning process. In many cases, patient partners were brought onto projects later in the process (due to funding timelines), which led to disappointment. This challenge was acknowledged by many, including CPN leadership and researchers, as a barrier to effective engagement. Most agree this was a key lesson learned for future engagement work. There was also a feeling among many Network members that there was insufficient pressure placed on the funded projects to fully comply with the engagement requirement: *“embedding [patient engagement] as an expectation and something that everyone was moving all along together on all the projects, that didn’t happen…that variability is something I’m not thrilled about”* (Interview #2022-11: CPN Staff/Leadership). Further, some projects struggled to determine how best to engage patient partners in their work, especially when the projects were more basic science in nature: “*the earlier, the more fundamental the research, the less sort of immediate impact, the less opportunity there is for a patient, for instance, you know to share what their experience is or to have a sense of where the patient experience fits into this….it’s too many steps removed….in that respect [patient engagement] is sort of difficult….maybe patient engagement needs to look different if you really want to do it for basic research”* (Interview #2020-11: Researcher).

Despite the variability in how successfully patients partnered with research teams, for several studies, these partnerships were viewed as leading to changes in the priorities or specific decisions made within the research process. One researcher noted: *“when I first started working with a patient partner, I had a hard time imagining what their role would be….[but through discussions] they gave us a fairly good idea for a direction to go with the research…I ended up developing a number of studies because of that conversation we had…it was a really impactful conversation”* (Interview #2020-14: Researcher). Another researcher highlighted how a conversation about recruitment strategies with their patient partner led to a change in approach which improved the success of their study, leading to a reduction in the time required to recruit participants.

### Enabling additional opportunities

An often-cited impact of patient engagement in the CPN was the creation of additional opportunities and activities beyond the Network. This occurred for both the Network as a whole, and for individuals within the Network. Connections made within the CPN opened the doors to other opportunities for many: *“I see that trend, that influence the CPN has…I see a lot of us patients engaged in patient engagement have been asked to participate [in] other studies that are being done now and they’re outside of the CPN per se. So, for example, I think that I am participating in about four research projects right now outside of CPN but with connections to CPN”* (Interview #2020-02: Patient Partner). In some cases, these opportunities grew out of the community and networks that were created within the CPN, but for some patient partners, their association with the CPN led to additional opportunities: *“[organization] approached me after they heard I was in the CPN to join them…so it just kind of snowballed”* (Interview #2022-01: Patient Partner). Interactions with patient partners and researchers also led to additional opportunities to work together on new projects for some. One researcher noted how a conversation with a patient partner led to a discussion of a new area of research they had not considered until then and an opportunity to work together on a new project: *“We thought, wow, here’s a really great idea for a project. Why don’t we go after that…and so the patient engaged with us for a new area of research that we’re now starting”* (Interview #2020-15: Researcher).

Several opportunities to influence policy and government also grew out of the CPN. The CPN was formed at a time when there was increasing attention on chronic pain, due in part to the opioid crisis. This positioned the CPN in a way to make a direct impact on policy. As a member stated in early interviews: *“I feel like it’s just kind of the perfect storm that’s happening within the policy community around opioids and there not being a go-to patient advocacy group for chronic pain”* (Interview #2018–09: CPN Staff/Leadership). The Opioid Response Team from Health Canada approached the CPN to identify and connect with patient partners to provide insights on the impact of the opioid crisis on their experiences [32]. Members highlighted how the federal government came to the CPN, in the absence of a national pain patient advocacy organization, to tap into the patient perspective on a number of topics: “*it was almost like government was coming to us and saying we need you to fulfill this advocacy gap that’s existing and even though that’s not necessarily why the Network exists, people within the Network were happy to see they were recognizing that gap and they were seeing CPN as an organization that could help them with those needs*” (Interview #2022-13: CPN Staff/Leadership). This perspective continued to unfold over time as the Network was described as a trusted group for individuals to reach out to: *“I think that, you know, externally our reputation is a trusted group of patient partners to work with…we’re building credibility and being respected for the knowledge we bring to the table”* (Interview #2020-02: Patient Partner).

This was most tangibly seen through the Canadian Pain Task Force, a national task force formed by the Federal Minister of Health to “*provide advice to Health Canada regarding evidence and best practices for the prevention and management of chronic pain*” [33]. While the CPN was not directly involved in the Task Force, numerous CPN members were, and many credited the CPN as having an impact on creating this opportunity: *“I think the fact they were engaged with CPN provided that platform, that diving board to get into this task force, because several of our members were involved in it. So, there was an influence there…the sheer fact that we existed opened that door”* (Interview #2022-05: Patient Partner).

### Promoting culture change

There are several indicators that point to the role that the CPN’s patient engagement strategy and the work that accompanied it played in shaping the culture within the Network and beyond. From the outset of the Network, the goal was to create a culture where *“we were all equal partners…if you were in the game, you were in the game [and] it didn’t matter if you were a patient or a researcher or an administrator or the nominated principal applicant or a co-applicant or whatever.”* (Interview #2018-12: CPN Staff/Leadership). CPN members had varied experiences establishing this level playing field, however, and this was identified as an early challenge as *“it takes time to build trust and relationships…it’s a huge culture shift”* (Interview #2018-14: Patient Partner). Although the Network did not have a full-time staff member in place to support patient engagement, it had strong leaders and facilitators of engagement through the co-chairs of the PE Committee and individuals within the governance committee, as well as a paid consultant with a strong background in engagement. This was identified as having an impact on the ability to change the culture of the network to support engagement, and on the overall functioning of the PE strategy: *“Having [these individuals] be there to support the work…has been really helpful….the patient engagement work within the Network would not have happened as well as it did without [them]”* (Interview #2022-10: Patient Partner).

By the end of the Network’s first phase, while challenges remained, many identified that there had been significant changes in the culture of the Network: *“everyone has kind of been on this journey together of learning about patient engagement, seeing the impact that patient engagement can have…it has become more and more embedded within the Network and within people’s personal values as well….they will continue to go on and do patient engagement because of everything they’ve seen and learned in the Network”* (Interview #2022-10: Patient Partner). Evidence of this shift of culture was also seen through the process for creating priorities for the second phase of CPN’s funding. During a 2019 priority setting meeting, the Steering Committee identified patient engagement as one of four key areas of interest and value for the next phase of the Network [25].

Members highlighted some specific areas where the CPN’s values and practices helped to shift the culture of engagement. One of these areas was related to providing honoraria to patient partners for their time and expertise. At the time of the CPN’s establishment, there was little guidance available on how to compensate patient partners, but this was a key tenet of the CPN’s work: *“…there was a large budget for honoraria for patient partners if they wanted to receive it. Which, given the fact that there was zero guidance from the funding agency about how to do that at the time, I would say that was innovative…”* (Interview #2022-13: CPN Staff/Leadership). CPN members went on to support the development of guidance in the area of patient partner compensation, along with other funded SPOR Chronic Disease Networks, contributing to further culture change around how patient partners are compensated within SPOR networks and beyond [27].

CPN products and resources also were credited with influencing the culture of engagement beyond the Network itself. The patient partner authorship and acknowledgement paper [24] has had a strong uptake in the research community, contributing to culture change: *“we have people contacting the Network saying, ‘we saw this article, can your patient partners and authors come and speak to our group about how to do this because we’re doing engagement but this next piece of co-authoring and producing knowledge, people hadn’t gotten there….”* (Interview #2022-11: CPN Staff/Leadership)*.*

During the CPN’s mandate, other research networks were formed, both in the area of pain and outside of pain. Some of these research networks learned from the engagement work of the CPN, further changing the culture of engagement in the research community: *“Since CPN started, there have been two other national research networks on pain that have been funded….both of them have a huge focus on engagement of people with lived experience and they’ve really learned from, and credited, the model we established at CPN”* (Interview #2022-11: CPN Staff/Leadership). Additionally, members noted that they heard other organizations talking about and referencing the CPN regarding patient engagement. This was further supported as members spread learnings from the CPN to other organizations that they were involved in: *“Every one of us made presentations to our own local organizations…I think we became some kind of a beacon in terms of a [patient engagement] organization and other individuals were interested….it starts with a few individuals and then it’s a big army and every one of those people can influence other people”* (Interview #2022-03: Patient Partner).

### Coping with experiences of living with chronic pain

Given the focus of this research network, all patient partners had personal lived experiences and/or caregiving experiences with pain. Throughout the interviews, numerous patient partners identified the challenges associated with participating in engagement activities while living with chronic pain (e.g., challenges attending meetings, memory issues associated with pain) and the benefits of working with others who understood these pain experiences. Beyond this, in the final round of interviews, a few patient partners reflected on how their work with the CPN helped them to cope with their chronic pain as it provided distractions and purpose and helped them to make connections with others: “*It’s helped me with my pain because I get out of myself, and I forget about it”* (Interview #2022-12: Patient Partner). This was felt strongly by some patient partners who expressed concerns about how the first phase of the Network ending would impact patient partners' experiences with pain: *“for five years we’ve been involved in something which is a coping mechanism for pain…I worry about where [patient partners] will be [if CPN ends]. Do they have something to fill the hole?”* (Interview #2022-07: Patient Partner).

## Discussion

While many organizations and research teams have sought to evaluate their engagement work, few have explicitly focused on assessing the impacts of engagement and collaborated with an external evaluation team to support this work. While this is a complex task, understanding the impacts of engagement is critical to moving patient engagement efforts forward. Researchers, funders and patient partners themselves want to know that the work that is being done is making a difference. In a study by Carroll et al. research scientists questioned the value of patient engagement and if it was justified as they felt there was insufficient evidence demonstrating impact [14]. While it’s clear the culture around patient engagement is shifting as it becomes more widely accepted and, in some cases, required by funders, being able to demonstrate impact is of key importance to gaining buy-in from all stakeholders.

Members credit the CPN for increasing the attention paid to patient engagement, demonstrating what effective patient engagement looks like, and providing members of the community with opportunities to learn and engage. Impacts were also seen beyond the Network. While attributing impacts to one research network is complex and difficult, members had a strong sense that the work of the Network had a lasting impact on the broader community and highlighted the importance of the influence they had not only on the science of pain, but also on the science of engagement.

Further, the evaluation results served as a learning and improvement tool both during the first phase of the CPN and for the CPN’s phase two application. Learnings that were uncovered through these phases were acted on in real time by the CPN leadership. For example, early evaluation results demonstrated that the impact of patient engagement on funded research projects was limited by the lack of buy-in and culture change from some research teams in the early stages. In response, strategies for engaging patient partners in research teams were adapted, which contributed to some of the more positive impacts that were traced later in the evaluation. Several of the key successes and challenges that were uncovered during the evaluation informed the Network’s phase two application. The importance of having strong staff support for engagement led to the inclusion of an engagement lead in the phase two budget, and the need for greater diversity in experiences and backgrounds amongst patient partners has been a focus for recruitment. As well, an equity, diversity and inclusion committee has been struck to support governance committees and work within the Network [23].

### Strengths and limitations

There were several strengths of this study. First, the collaboration with an external evaluation team (PPEC) provided a level of independence and ‘safe space’ to support and facilitate the evaluation. While the goals and objectives of the evaluation were co-developed with the CPN, in particular the PE Committee, all data collection and analysis were conducted independently promoting objectivity and reducing common biases that have been identified as limitations in similar evaluations conducted internally [6]. The longitudinal nature of the evaluation also provided opportunities for the CPN to learn from the evaluation results and adapt their work in real time, and provided opportunities to see impacts unfolding over the course of the Network’s evolution. Our decision to conduct interviews over the lifespan of the Network allowed for unanticipated impacts to emerge, and for a more in-depth understanding of the impacts than we would have achieved through surveys alone.

This study is not without limitations. The evaluation sought to connect with a wide range of stakeholders who were involved with the CPN, however, we were not able to reach all members during the interview phases. It may be that those individuals who agreed to participate had different experiences and perspectives on the CPN’s patient engagement activities than those who did not. Generally, CPN funded researchers were less likely to agree to be interviewed than CPN staff and patient partners. Further, CPN funded researchers were only invited to participate in one round of interviews, unless they met other inclusion criteria (e.g., were a member of the PE Committee or were a Committee co-chair), and thus had fewer opportunities to agree to participate in interviews. As a result, their perspectives are not included in as much depth in our analysis and their insights may have differed from those who did participate. Further, the narrower focus on documenting the experiences of CPN members to assess impact, excluded outreach to others in the pain research community, thus limiting assessment of broader impact to CPN members’ perceptions. Further research could be conducted to explore non-CPN members’ perspectives on these impacts.

## Conclusions

The Chronic Pain Network is built on strong clinician, researcher and patient partnerships, with an ongoing commitment to greater patient engagement as a priority. Although not without challenges, the engagement strategy and its implementation has had demonstrable impact on the people involved in the Network, policies, the Network itself and the funded research projects. The impacts were also seen more broadly on the pain research community in Canada, and on the broader culture of patient engagement in research. Understanding the impacts of patient engagement on national health research networks and in other contexts is an emerging area of research that holds promise for improving our understanding of how these types of networks can contribute to building research communities that fully support and appreciate the value of patient engagement in research and research governance. Our findings demonstrate the value of having patient partners engaged in all aspects of a research network and should serve as encouragement to others taking on similar work.

### Supplementary Information

**Additional file 1.** Interview Guides.

**Additional file 2.** GRIPP-2 Long Form.

## Data Availability

The datasets generated during the current study are not publicly available but may be available from the corresponding author on reasonable request.

## Abbreviations

CIHR: Canadian Institutes of Health Research
CPN: Chronic Pain Network
NIHR: National Institute for Health and Care Research
PCORI: Patient-Centered Outcomes Research Institute
PE: Patient engagement
PIHCI: Primary and Integrated Health Care Innovations
PPEC: Public and Patient Engagement Collaborative
SPOR: Strategy for Patient-Oriented Research
