# Supplementary material for: Evaluating the impacts of patient engagement on a national health research network: results of a case study of the Chronic Pain Network
Source: Res Involv Engagem. 2023 Aug 30;9:73. doi: 10.1186/s40900-023-00491-w (PMC10466858; doi:10.1186/s40900-023-00491-w)
Supplement: Supplementary file 1 — Additional file 1. Interview Guides. [file 40900_2023_491_MOESM1_ESM.pdf]

Supplementary File 1: Interview Guides

ROUND ONE

PATIENT PARTNER INTERVIEW GUIDE

1. To begin, could you please tell me a little bit about your background?
    a. What past experiences do you have with patient engagement?
    b. What is your professional background?
    c. What other roles do you carry out while acting as a patient partner for the CPN?
    d. Given that the focus of this network is chronic pain, we're interested to know the background of the group (researchers and patients) in this area. Could you please describe your background in the area of chronic pain?

2. What do you think patient engagement means?

3. How were you recruited to participate in the CPN?
    a. How did you first learn about this network?
    b. When did you join the network?
    c. Why did you decide to get involved?
    d. What was the process of joining the network as a Patient Partner?

4. What is your role in the Network?
    a. What roles do you play in the governance of the network?
    b. Do you work with any of the CPN funded research teams as a patient partner?
    c. What other roles do you have in the network?

5. Have you ever had a discussion with the CPN leadership about your role in the network? If yes, what was covered in that discussion?
    a. Has the role changed from how it was defined originally (if yes, how)?

6. What information was given to you (if any) about the roles of other members in the network?
    a. Do you understand the roles others play?

7. How prepared do you feel to contribute to this network?
    a. What training, support or past experiences have helped you feel prepared?
    b. What additional training or support would be helpful to receive?

8. What were your expectations for this network in regards to the patient engagement component?

9. We all bring different perspectives to the group that influence how we go about our work. Whose perspectives would you say you're representing (or trying to represent) in the network?

10. When thinking about the committees you're a part of and the CPN as a whole, what are the different types of perspectives that you think are being shared during these activities?

11. Now that you have been part of the network since______, have your experiences matched your original expectations for joining the network? [If not, why not?]

12. What are some examples of how you contribute to patient engagement in the network?

13. How does the network leadership support your participation in the CPN?

14. You're a member of the _______ committee(s), correct? I'd like to spend some time to discuss your work with these committees. When answering the following questions please consider how each committee functions, and I'd encourage you to provide specific examples from the different committees, as we know each committee may run differently.
    a. How are meetings run?
    b. What do typical conversations look like?
    c. How do the patient partners and researchers work together? Probe re: power dynamics

15. Do you feel comfortable sharing your perspective during meetings, even if you think it might not be shared by others in the group?

16. How would you describe the relationship between researchers and patient partners on the committees?
    a. Do you feel like the views and perspectives of all committee members are considered equally? Why or why not?

17. How much influence would you say you have on various parts of the committee's work?
    a. [If they indicate that they are having influence] How do you know that you are having any influence?

18. What is going well with the committees?

19. Is there any way your experience as committee member can be improved?

20. [Ask if co-chair] Please describe the process of co-chairing a committee meeting with a researcher?
    a. How do you prepare for the meeting?
    b. How do you conduct the meeting?
    c. What is going well? What can be improved?

21. Tell me about your experience with ____________________. (Research Project)
    a. What is your role on the project?
    b. How frequently do you engage with the research team?
    c. What's going well with this partnership?
    d. What could be improved?
    e. Do you feel like you're making an impact on the research project? [If yes, how do you know this? If no, how do you know this?]

22. Sometimes there are challenges when researchers, patients and other stakeholders work together. These might include things like finding a convenient time to meet or clearly communicating with each other.
    a. What have been the biggest challenges for you, so far, working with CPN?
    b. What have been the biggest successes for you, so far, working with CPN?
    c. Do you have any suggestions for improvement?

23. What have you contributed to the CPN, and how do you think this has this made a difference?

24. Is there anything else that hasn't been covered that you had hoped to discuss during our call?

RESEARCHERS / STAFF / LEADERSHIP INTERVIEW GUIDE

1. To begin, could you please tell me a little bit about your background?
    a. What past experiences do you have with patient engagement?
    b. What is your professional background?
    c. Given that the focus of this network is chronic pain, we're interested to know the background of the group (researchers and patients) in this area. Could you please describe your background in the area of chronic pain?

2. What do you think patient engagement means?

3. Have you ever worked with a patient/caregiver partner on a research project/network/collaborative prior to this one?

4. How were you recruited to participate in the CPN?

5. What is your role in the Network?
    a. What roles do you play in the governance of the network?
    b. Do you work with any of the CPN funded research teams?
    c. What other roles do you have in the network?

6. How prepared do you feel to work with patient partners in this network?
    a. Have you had training on patient engagement?
    b. What training, support or past experiences have helped you feel prepared?
    c. What additional training or support would be helpful to receive?

7. What steps do you or others on the research team take to support patient partners' participation in this network?

8. How prepared do you feel to contribute to this network?
    a. What training, support or past experiences have helped you feel prepared?
    b. What additional training or support would be helpful to receive?

9. What were your expectations for this network in regards to the patient engagement component?
    a. What did you hope to contribute to the network?

10. We all bring different perspectives to the group that influence how we go about our work. Whose perspectives would you say you're representing (or trying to represent) in the network?

11. When thinking about the committees you're a part of and the CPN as a whole, what are the different types of perspectives that you think are being shared during these activities?

12. Now that you have been part of the network since______, have your experiences matched your original expectations for joining the network? [If not, why not?]

Questions for those who are members of committees

13. You're a member of the _______ committee(s), correct? I'd like to spend some time to discuss your work with these committees. (It on multiple committees…)When answering the following questions please consider how each committee functions, and I'd encourage you to provide specific examples from the different committees, as we know each committee may run differently.
    a. How are meetings run?
    b. What do typical conversations look like?
    c. How do the patient partners and researchers work together? *Probe re: power dynamics*

14. How do the patient partners participate in the committee? How do you support this participation?

15. What is going well with the committees?

16. Is there any way your experience as committee member can be improved?

17. [Ask if a part of 2 or more committees] How do your experiences compare across the committees you sit on?

Questions for all interviewees

18. What is your personal view regarding some of the potential barriers or challenges to patient engagement in the research network context?

19. How much influence would you say the patient partners have had on various parts of the network?
    a. Do you feel as if they have had sufficient influence?

20. What have patient partners contributed to this network thus far, and how has this made a difference?
    a. In your opinion, have the insights and comments of the patient/caregiver partners impacted the decisions of the network?

21. Do you feel you and the network listened to and absorbed the input from the patient/caregiver partners to date?

22. Sometimes there are challenges when researchers, patients and other stakeholders work together. These might include things like finding a convenient time to meet or clearly communicating with each other.
    a. What have been the biggest challenges for you, so far, working with CPN?
    b. What have been the biggest successes for you, so far, working with CPN?
    c. Do you have any suggestions for improvement?

23. What have you contributed to the CPN, and how do you think this has this made a difference?
    a. Are there sufficient opportunities for you to contribute to this network?
    b. In what ways could this be improved?

24. Is there anything else that hasn't been covered that you had hoped to discuss during our call?

ROUND TWO

PATIENT PARTNER INTERVIEW GUIDE

Questions for those who did not participate in Round 1

1. To begin, could you please tell me a little bit about your background?
    a. What past experiences do you have with patient engagement?
    b. What is your professional background?
    c. What other roles do you carry out while acting as a patient partner for the CPN?
    d. Given that the focus of this network is chronic pain, we're interested to know the background of the group (researchers and patients) in this area. Could you please describe your background in the area of chronic pain?

2. How long have you been a part of the network?

3. How would you describe patient engagement to someone else? What does that term mean to you?

4. How did you get involved with the CPN? What was the recruitment process like?

5. What is your role in the Network?
    a. Which committee are you a part of?
    b. Do you work with any of the CPN funded research teams as a patient partner?
    c. What other roles do you have in the network?

6. How prepared did you feel to contribute to this network?
    a. What training, support or past experiences have helped you feel prepared?
    b. What additional training or support would be helpful to receive?

7. What were your expectations for this network in regard to the patient engagement component?

8. Now that you have been part of the network since______, have your experiences matched your original expectations for joining the network? [If not, why not?]

9. What are some examples of how you have been able to contribute to the network?
    a. What are factors that support your engagement in the network?
    b. What are factors that inhibit your engagement in the network?
    c. Have you gained any new skills or improved your skill level through your work in the network?

Questions for those who did participate in Round 1

10. During the last interview you had explained your experience with patient engagement and chronic pain. Has anything changed over the past year or would you like me to know anything else?

11. Last year you defined patient engagement as _______________________. Do you think about patient engagement in the same way now or would you define it differently today?

12. Has your role in the network changed since we last spoke?
    a. Which committee are you a part of?

   b. Do you work with any of the CPN funded research teams as a patient partner?
   c. What other roles do you have in the network?

13. Based on your experience in these network roles, what advice would you give to a new member who was starting a similar role in the network?

14. Over the past year has CPN provided any new trainings and/or supports for your role(s)?

15. Has the network has matured, have your expectations for its' patient engagement component changed? If yes, how so?

16. What are some examples of how you have been able to contribute to the network over the last year?

Questions for all interviewees

You're a member of the _______ committee(s), correct? I'd like to spend some time to discuss your work with these committees. When answering the following questions please consider how each committee functions, and I'd encourage you to provide specific examples from the different committees, as we know each committee may run differently.

17. Tell me about your committee.
    a. purpose/scope?
    b. Meetings style?
    c. Conservational style?
    d. Dynamics between members?

18. Do you feel comfortable sharing your perspective during meetings, even if you think it might not be shared by others in the group?

19. How would you describe the relationship between researchers (or other groups) and patient partners on the committees?

20. How much influence would you say you have on various parts of the committee's work?
    a. [If they indicate that they are having influence] Can you think of an example you could share of how you've influenced the committee? (probe for specific stories, examples)

21. Reflecting on your experiences with the committee, can you tell me about what is going well and how your experience could be improved?

22. [Ask if a part of 2 or more committees] How do your experiences compare across the committees you sit on?
    a. Are the committee meetings conducted in different ways? [If so, do you prefer the way that some committee meetings are run over others?]

23. [Ask if co-chair] Please describe the process of co-chairing a committee meeting with a researcher?
    a. How do you prepare for the meeting?
    b. How do you conduct the meeting?
    c. What is going well? What can be improved?

24. If there is a call for renewals and the network is renewed, should anything be changed about the co-chair structure?

Questions for those who indicated they are involved in CPN funded research projects as a patient partner

25. How did you get involved with the research project?
    a. What aspects of the project are you involved in?

26. What information did the research team give about your role on the project?

27. What are some examples of tasks or activities you completed as part of your research project role?

28. How do you typically communicate with the research team (zoom, telephone, in-person, etc.)?
    a. How frequently do you communicate with the team?
    b. Do you feel comfortable interacting with research team and voicing your opinion?

29. What are some examples of things that are going well with your research partnership? What are some examples of things that could be improved with your research partnership?

30. Can you think of an example of how you've made an impact on the research project? (If they don't think they have made an impact – probe as to why)

31. What have you learned as a result of this partnership?

32. If the network was going to start again, what changes would you make to how these research partnerships were set up and how they function?

Questions for all interviewees

33. Sometimes there are challenges when researchers, patients and other stakeholders work together. These might include things like finding a convenient time to meet or clearly communicating with each other.
    a. What have been the biggest challenges for you, so far, working with CPN?
    b. What have been the biggest successes for you, so far, working with CPN?
    c. Do you have any suggestions for improvement?

34. At this point in the CPN's timeline, what would you say has been the biggest impact of the network?

35. What have you contributed to the CPN, and how do you think this has this made a difference?

36. We don't know at this stage if there will be a call for renewals for these networks, but if there was and if the CPN applied for renewal – what do you think should stay the same? What do you think should be changed?

37. Is there anything else that hasn't been covered that you had hoped to discuss during our call?

RESEARCHER INTERVIEW GUIDE

1. To begin, could you please tell me a little bit about your background?
    a. What past experiences do you have with patient engagement?
    b. Can you describe your experience with chronic pain research?

2. How would you describe patient engagement to someone else? What does that term mean to you?

3. Could you give me a quick overview of your CPN funded study?

4. Can you please describe to me how you first decided to engage a patient partner in your research?
    a. Why were you interested in working with a patient partner?
    b. What were your expectations for the partnership?

5. How did you connect with the CPN patient partner to work on your research project?
    a. Did CPN assign a patient partner or help you find one? If not, how did you recruit a patient partner?
    b. How did this recruitment process unfold?

6. How prepared did you feel to work with patient partners?
    a. Have you had training on patient engagement?
    b. What information did the CPN provide you? Did CPN provide training or resources?
    c. What training, support or past experiences have helped you feel prepared?
    d. What additional training or support would be helpful to receive?

7. Tell me about your initial meeting with the patient partner(s).
    a. How was the patient partner orientated to the project?
    b. Was the patient partner role discussed?
    c. What expectations did the patient partner express for your partnership? Did this match with the expectations you had for their role on the project?

8. How do you typically communicate with the patient partners (zoom, telephone, in-person etc.)?
    d. How frequently are you in contact with patient partners?
    e. What is the style of your communication (i.e., informal vs formal)?
    f. What type of info is usually conveyed (e.g., project updates, task updates)?

9. What aspects of the project has the patient partner been involved in? How have they been involved (e.g., providing advice, participating in the research activities (e.g., analysis, tool development etc.), communicating research results).

10. Can you provide some concrete examples of how the patient partner contributed to this project?
    a. Where there any times that you had hoped to include the patient partner and were not able to do so? What were the barriers?

11. Is this patient partner still engaged in your research project? If not, when did this partnership end?

12. Did involving patient partners in your research go as planned?
    b. What worked out the way you expected? What happened differently?
    c. What would you do differently if you could start this process again?

13. Reflecting on the project so far, what would you say the impact of working with a patient partner has been?
    a. What value (if any) is created by involving patient partners in research?
    b. Do you work differently? Does your team work differently?
    c. Do you think about the research process differently?

14. What are some examples of things that are going well with your research partnership?

15. What are some examples of things that could be improved with your research partnership?

16. What have you learned as a result of this partnership?

17. Would you work with patient partners on a research project again? Why or why not? If not → what could be done to make this easier/more impactful?

18. If the network was going to start again, what changes would you make to how these research partnerships were set up and how they function?

Questions for interviewees who are a member of CPN committees

You're a member of the _______ committee(s), correct? I'd like to spend some time to discuss your work with these committees, and to learn more about how patient partners are engaged on the committee.

19. Tell me about your committee.
    a. purpose/scope?
    b. Meetings style?
    c. Conservational style?
    d. Dynamics between members?

20. Reflecting on the role of the patient partner on the committee…how would you describe their participation?
    a. Have the patient partners shared their personal experiences as a patient or caregiver?
    b. How are patient partners supported to encourage them to share their perspective?

21. Do you feel you and the network listened to and absorbed the input from the patient/caregiver partners to date?

22. What is going well with the committees?

23. Is there any way your experience as committee member can be improved?

24. [Ask if a part of 2 or more committees] How do your experiences compare across the committees you sit on?

25. If there was a call for renewal for the network, should any changes be made to how patient partners are integrated into the committees?

26. [Ask if co-chair] Please describe the process of co-chairing a committee meeting with a patient partner.

Questions for all interviewees

27. At this point in the CPN's timeline, what would you say has been the biggest impact of the network?

28. We don't know at this stage if there will be a call for renewals for these networks, but if there was and if the CPN applied for renewal – what do you think should stay the same? What do you think should be changed?

29. Is there anything else that hasn't been covered that you had hoped to discuss during our call?

ROUND THREE

PATIENT PARTNER INTERVIEW GUIDE

1. To begin, could you please tell me a little bit about your role(s) within the Chronic Pain Network and specifically if there have there been any changes to your role in the past year or so?

2. Thinking back on your initial motivations for joining the Chronic Pain Network…why did you initially want to join/get involved? What did you hope to achieve? Have your motivations changed over time?
    a. Now that the network is at the end of its first mandate, do you feel like you've been able to achieve what you set out to do? Why or why not?

3. Now I'd like to ask you some questions about CPN's approach to engagement and the supports for PE that were put in place within Network…
    a. Thinking broadly, how would you describe the CPN's approach to patient engagement to someone who wasn't familiar with the Network?
    b. Again, thinking broadly, how would you describe the supports that were put in place for patient engagement in the Network? Did these supports change over time?
    c. Overall, what do you think worked well about how patient engagement was structured and supported? What improvements could have been made to how patient engagement was structured and supported?

4. Sometimes there are challenges when groups of people work together. What challenges or negative impacts have you experienced as a result of participating as a patient perspective partner in the CPN?
    a. Were these challenges addressed in any way in your view?
        i. If yes, how? If no, how could they have been addressed?
    b. What supports could be put in place to prevent this from happening in the future?

5. What impacts has engaging as a patient perspective partner in the CPN had on you as an individual?

6. When you think about patient engagement within the CPN, would you say that it's embedded within the organization or is it dependent on a few individuals being champions for the work? Would patient engagement be sustainable within the CPN if the key leaders/champions left the organization?

7. How would you describe the impact that patient engagement has had on the work and activities of the Chronic Pain Network?
    a. What changed because patient perspective partners were engaged? Can you provide any specific examples?

8. From previous interviews, we know that some individuals felt that the CPN's work shifted to include some advocacy-related work in the area of pain to address a gap in this area.
    a. How do you view patient engagement in relation to advocacy work? How are they different? How are they similar or complementary to each other?
    b. How well did CPN balance their advocacy and patient engagement activities? What went well? What could have been changed or approached differently?

9. Can you think of any examples of how the CPN's approach to or outputs from its patient engagement work has impacted the broader research or pain community, or the health system?
    a. Has your work with CPN led to any additional opportunities outside of the network?

   b. Are other groups aware of CPN's patient engagement work? How do you know this?
   c. Can you think of any examples of how patient engagement in CPN funded research has had an impact on the research community?

10. As this round of funding for CPN is wrapping up, what do you think the main lessons learned are from the approach CPN took to patient engagement?
    a. What could other groups learn from the CPN?
    b. What should be replicated?
    c. What should be changed?

11. Has your perspective on patient engagement changed during your time with CPN? How so?

12. Is there anything else that you want to share about the CPN's patient engagement strategy at this time?

CPN LEADERSHIP, RESEARCHER INTERVIEW GUIDE

1. To begin, could you please tell me a little bit about your role(s) within the Chronic Pain Network? Have there been any changes to your role in the past year or so?

2. Thinking back on when you first got involved with the CPN, what were your goals or hopes for patient engagement in the network? Have your goals/hopes for patient engagement within the CPN shifted or changed over time?
   a. Now that the network is at the end of its first mandate, do you feel like you've been able to achieve what you set out to do? Why or why not?

3. Now I'd like to ask you some questions about CPN's approach to engagement and the supports for PE that were put in place within Network…
   a. Thinking broadly, how would you describe the CPN's approach to patient engagement and the supports that were in place to someone who wasn't familiar with the Network?
   b. Overall, what do you think worked well about how patient engagement was structured and supported? What improvements could have been made to how patient engagement was structured and supported?

4. Sometimes there are challenges when groups of people work together. What challenges or negative impacts have you experienced as a result of engaging with patient partners in the CPN?
   a. Were these challenges addressed in any way in your view?
      i. If yes, how? If no, how could they have been addressed?
   b. What supports could be put in place to prevent this from happening in the future?

5. What impacts has engaging with patient partners in the CPN had on you as an individual?

6. When you think about patient engagement within the CPN, would you say that it's embedded within the organization or is it dependent on a few individuals being champions for the work? Would patient engagement be sustainable within the CPN if the key leaders/champions left the organization?

7. How would you describe the impact that patient engagement has had on the work and activities of the Chronic Pain Network or how the work was carried out?

    b. What changed because patient partners were engaged? Can you provide any specific examples?

8. From previous interviews, we know that some individuals felt that the CPN's work shifted to include some advocacy-related work in the area of pain to address a gap in this area.
    a. How do you view patient engagement in relation to advocacy work? How are they different? How are they similar or complementary to each other?
    b. How well did CPN balance their advocacy and patient engagement activities? What went well? What could have been changed or approached differently?

9. Can you think of any examples of how the CPN's approach to or outputs from its patient engagement work has impacted the broader research or pain community, or the health system?
    a. Has your work with CPN led to any additional opportunities outside of the network?
    b. Are other groups aware of CPN's patient engagement work? How do you know this?
    c. Can you think of any examples of how patient engagement in CPN funded research has had an impact on the research community?

10. As this round of funding for CPN is wrapping up, what do you think the main lessons learned are from the approach CPN took to patient engagement?
    a. What could other groups learn from the CPN?
    b. What should be replicated?
    c. What should be changed?

11. Has your perspective on patient engagement changed during your time with CPN? How so?

12. Is there anything else that you want to share about the CPN's patient engagement strategy at this time?
